# Investigation of the long-term healing response of the liver to boiling histotripsy treatment in vivo

**DOI:** 10.1038/s41598-022-18544-7

**Published:** 2022-08-24

**Authors:** Jeongmin Heo, Chanmin Joung, Kisoo Pahk, Ki Joo Pahk

**Affiliations:** 1 Center for Bionics, Biomedical Research Institute, Korea Institute of Science and Technology (KIST), Seoul, Republic of Korea; 2 Institute for Inflammation Control, Korea University, Seoul, Republic of Korea; 3 Department of Nuclear Medicine, Korea University College of Medicine, Anam-dong 5-ga, Seongbuk-gu, Seoul, 02841 Republic of Korea; 4 Department of Biomedical Engineering, Kyung Hee University, 1732 Deogyeong-daero, Giheung-gu, Yongin-si, Gyeonggi-do 17104 Republic of Korea

**Keywords:** Biomedical engineering, Therapeutics

## Abstract

Boiling histotripsy (BH) is a promising High-Intensity Focused Ultrasound technique that can be employed to mechanically fractionate solid tumours. Whilst studies have shown the feasibility of BH to destroy liver cancer, no study has reported on the healing process of BH-treated liver tissue. We therefore extensively investigated the evolution of the healing response of liver to BH in order to provide an insight into the healing mechanisms. In the present study, 14 Sprague Dawley rats underwent the BH treatment and were sacrificed on days 0, 3, 7, 14, and 28 for morphological, histological, serological and qPCR analyses. The area of the treated region was 1.44 cm^2^ (1.2 cm × 1.2 cm). A well-defined BH lesion filled with coagulated blood formed on day 0. A week after the treatment, fibroblast activation was induced at the treatment site, leading to the formation of extracellular matrix structure (ECM). The ECM was then disrupted for 7 to 28 days. Regenerated normal hepatocytes and newly formed blood vessels were found within the BH region with the absence of hepatic fibrosis. No significant morphological, histological and genetic changes around the BH lesion occurred. These results suggest that BH could be a safe and promising therapeutic tool for treating solid tumours without inducing any significant adverse effect such as the formation of liver fibrosis.

## Introduction

High intensity focused ultrasound (HIFU) is a non-invasive and non-ionising ultrasonic technique that has been used to thermally necrose solid tumours without damaging surrounding tissue. HIFU involves focusing an intense ultrasound beam into a small region of interest within the body. This results in localised tissue heating (> 55 °C) and protein denaturation at the HIFU focus, which can cause irreversible cell death through thermal coagulative necrosis^1^. HIFU thermal ablation has received FDA (U.S. Food and Drug Administration) approval for the treatments of uterine fibroids, pain palliation of bone metastases, osteoid osteoma, benign prostatic hyperplasia, prostate cancer and essential tremor^2–5^. The majority of heat deposition occurs in the HIFU focal volume with temperatures outside this focus being kept at noncytotoxic levels. The extent of necrotised tissue is therefore spatially confined to the HIFU focal region whilst sparing the intervening tissue^6^. The size and shape of the HIFU focal region is typically an ellipsoid or a cigar shape with 2–3 mm in lateral width and 8–10 mm in axial length along the wave propagation direction^7^.

In addition to HIFU thermal ablation, recent studies have clearly demonstrated the feasibility of employing HIFU to mechanically fractionate or break down solid tumours at the HIFU focus via acoustic cavitation. This promising HIFU technique known as boiling histotripsy (BH) which uses a number of millisecond long HIFU pulses containing shocks with high peak positive (*P*_+_) and negative (*P*_−_) pressures at the HIFU focus can produce violent acoustic cavitation resulting in mechanical tissue fractionation^8–10^. Tissue debris remaining inside a BH lesion is likely to be absorbed as part of the physiologic healing response whereas HIFU thermal lesion becomes fibrous scar tissue^8^. Whilst BH has not been yet approved by FDA, numerous studies have shown that BH can be used to mechanically destroy different types of soft tissues (e.g., heart, kidney, liver and prostate) and cancer cells (e.g., renal cell carcinoma, MDA-MB-231 human breast cancer, B16 melanoma) without causing any significant thermal damage to the boundary of a BH lesion induced^9–18^. The overall shape of a BH lesion is tadpole like consisting of a head and a tail with the head closest to the HIFU source^17,19^. Localised shockwave heating-induced boiling bubbles and shock scattering-produced subsequent cavitation clouds are, respectively, responsible for the creations of the tail and the head of a BH lesion^17,20,21^. Detailed mechanisms of BH can be found elsewhere^9,17,19,20,22^. In case of HIFU thermal ablation, blood perfusion can lead to undertreatment of a targeted tissue due to heat sink effect, and thus normal tissue surrounding the HIFU focus can possibly be ablated via thermal diffusion^23,24^. On contrary, since BH treatment is not greatly affected by heat perfusion via blood flow and thermal diffusion^11,25^, BH can produce a sharply demarcated margin between treated (fractionated tissue) and untreated (intact) regions with a transition distance of less than a cell length of the order of a micrometre. Furthermore, the process of BH treatment can be monitored using conventional B-mode ultrasound technique, because bubbles are highly reflective to ultrasound. During a BH exposure, for instance, boiling bubbles and cavitation clouds can appear as a hyperechoic region whereas a BH lesion is visible as a hypoechoic region on B-mode images in vivo^12^. In addition, recent studies have reported that BH can also induce a stronger immune response than that obtained with HIFU thermal ablation as more non-denatured antigenic proteins which enhance immune reactions can be released after BH exposure^18,26,27^. Due to the aforementioned advantages of BH over HIFU thermal ablation, BH has now gained significant interests in the field of HIFU for treating solid tumours.

Liver cancer is the sixth most common cancer and the fourth leading cause of cancer-related death worldwide with 841,080 new liver cancer cases diagnosed in 2018^28^. According to the liver cancer treatment guidelines provided by the Barcelona Clinic Liver Cancer (BCLC) staging system^29^ and the Asia Pacific Association for the Study of the Liver (APASL)^30^, patients with early-stage liver cancer (i.e., BCLC-0) can be treated with surgical liver resection or percutaneous ablation through radiofrequency or microwave, or trans-arterial radioembolization. These methods are invasive or minimally invasive. Whilst hepatic resection (hepatectomy) has been the mainstay of the treatment for early-stage liver cancer, the surgical procedures involved can however lead to certain complications such as bleeding, infection, blood clots and pneumonia. Noninvasive mechanical destruction of liver cancer using BH could potentially be a promising alternative or additional treatment option to minimise or even prevent these surgical risks. For BH to be used clinically, however, the efficacy and safety of BH including potential adverse tissue effects after BH treatment should be carefully evaluated. In fact, a large number of BH studies have clearly demonstrated the efficacy of BH for mechanically destroying solid tumours at the HIFU focus^9–18^, whereas no study has reported on the healing and repair process of the BH-treated tissue. This study therefore aims to provide an insight into the healing mechanisms and pathways triggered by BH through the observation of a long-term wound healing response of the liver to BH exposure.

## Results

**Figure 1:**
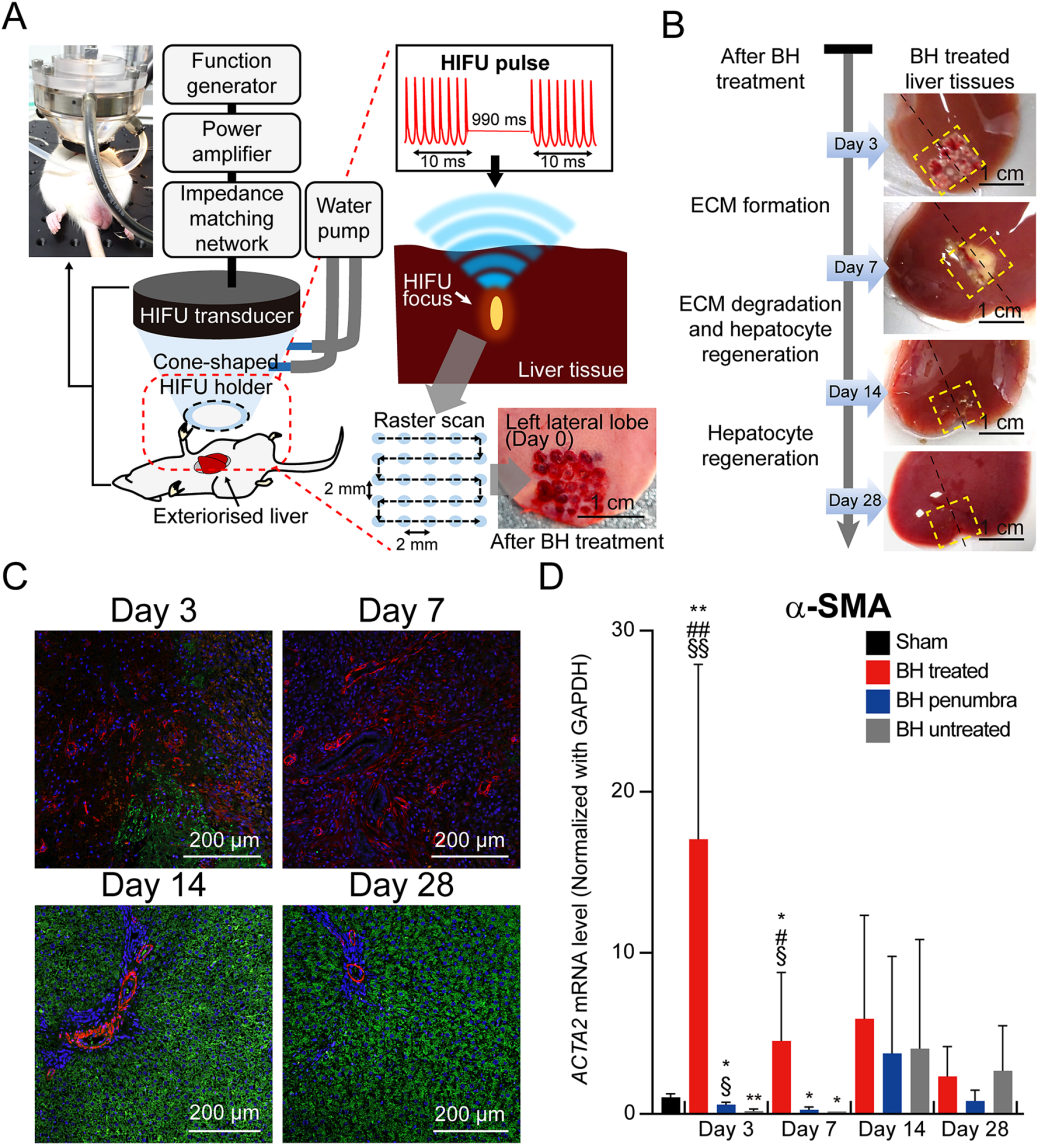
Experimental method and regeneration of hepatocytes with degradation of fibroblasts after the BH treatment. (**A**) A schematic diagram of the experimental setup for performing boiling histotripsy in rat’s liver in vivo*.* The HIFU transducer was placed on the exteriorized liver tissue and a 10 ms-long HIFU pulse with a pulse repetition frequency of 10 Hz was used. The HIFU focus was moved by raster scan resulting in the production of a number of BH lesions in the liver over the area of 1.44 cm^2^ (1.2 cm × 1.2 cm). The photo of the in vivo experimental setup was adapted from Supplementary Fig. S5. The photo of the BH treated liver was adapted from Supplementary Fig. S1. (**B**) Morphological observation of the liver tissue on days 3, 7, 14 and 28 after the BH treatment. The yellow outlined boxes and black dot lines indicate BH-treated regions and cross section lines for histological observation. (**C**) Representative IHC staining images of fibroblasts and hepatocytes in the cross-sectioned BH-treated liver tissues. Hepatocytes were immunostained with Asgr1 antibody (green), and fibroblasts were immunostained with α-SMA antibody (red). Nuclei were counterstained with DAPI (blue). (Scale bar, 200 µm; magnification, 100 ×) (**D**) Expression mRNA levels of α-SMA. *n* = 5 for Sham, *n* = 3 for Day 3 and 7, *n* = 4 for Day 14, and *n* = 5 for Day 28. Data are presented as means ± standard deviation (SD) of individual values. One-way analysis of variance (ANOVA) followed by post-hoc Tukey’s test was used. ^*§*^*p* < *0.05 vs. BH untreated of each time point*, ^*§§*^*p* < *0.01 vs. BH untreated of each time point*, ^*#*^*p* < *0.05* vs. *BH penumbra of each time point*, ^##^*p* < *0.01* vs. BH penumbra of each time point, *p < *0.05 vs. sham* and *p < *0.01 vs. sham*.

In the present study, a total number of 19 Sprague–Dawley (SD) rats were used to investigate a long-term wound healing process of the liver after BH treatment. Five SD rats were in sham group whilst 14 rats underwent BH experiments. A 2 MHz HIFU transducer coupled with a customised cone-shaped holder filled with degassed and deionised water was then placed on the animal’s exteriorised liver (Fig. 1A). During the experiment, a 10 ms-long BH pulse with peak positive pressure *P*_+_ of 85 MPa, peak negative pressure *P*_−_ of −14 MPa at the focus, a pulse repetition frequency of 1 Hz and a duty cycle of 1% was used to mechanically fractionate liver tissue. A total number of 25 BH lesions were created in the liver over the area of 1.4 cm^2^ (1.2 cm × 1.2 cm, as shown in Fig. 1A).

### Regeneration of hepatocyte and degradation of fibroblast after BH treatment

Morphological changes of the liver tissue treated with BH over time (i.e., day 3, 7, 14 and 28) are shown in Fig. 1B (i.e., the areas indicated within the yellow dashed lines). On day 3, the formation of early ECM was clearly observed in the BH-treated liver tissue and scar tissue subsequently formed over the area of approximately 0.4 cm^2^ (0.7 cm × 0.57 cm) on day 7. This area shrinkage (from 1.4 cm^2^ on day 3 to 0.4 cm^2^ on day 7) is most likely to be due to wound contraction as a part of the healing process^31,32^. The length and width of the scar tissue were measured using a digital caliper, and the area was calculated by multiplying the length by the width. In the early stage of the normal wound healing process, fibroblasts secrete collagen which is the main component of ECM. During the experiments, significant amounts of α-smooth muscle-actin (α-SMA) expressing active fibroblasts were observed on days 3 and 7, as shown in Fig. 1C. Simultaneously, the expression of hepatocyte marker (Asialoglycoprotein receptor 1, Asgr1) was depleted at the BH-treated site (Fig. 1C). The high expression of activated fibroblast (α-SMA) was also confirmed by qPCR analysis (Fig. 1D). On day 3, the mRNA level of α-SMA in the BH-treated region significantly increased compared to the sham group and the BH-untreated region, which then gradually decreased over days 7 to 28. After 14 and 28 days of the BH exposure, a large population of hepatocytes was clearly observed along with the appearance of portal vein within the BH-treated region (Fig. 1C). In addition, no significant scar tissue was found on day 28 (Fig. 1B). It is worth noting that the level of α-SMA of the BH-penumbra was similar to that of the BH-untreated region (Fig. 1D). These results can imply that BH can lead to highly localised biological effects at the treatment site (i.e., HIFU focus).

### Histological observation on the process of proliferation and maturation after BH exposure

**Figure 2:**
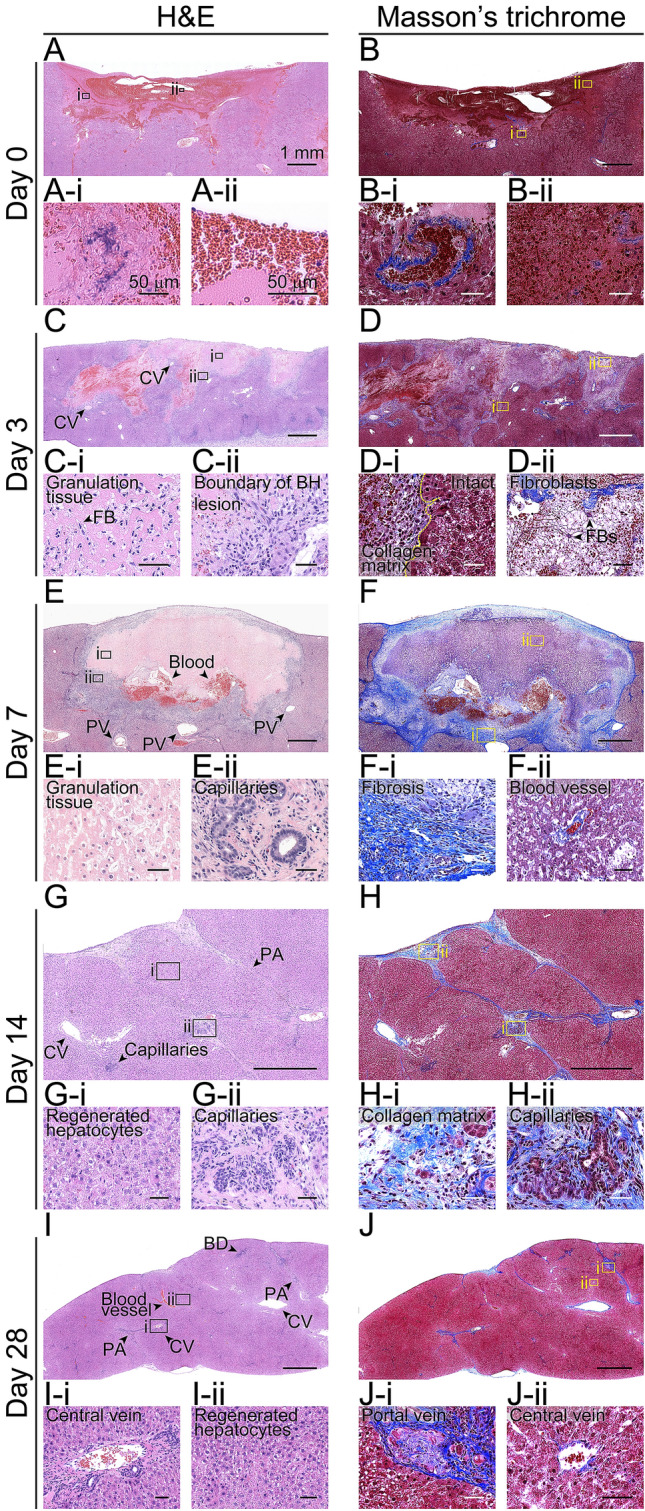
Cross-sectional images of the BH-treated liver tissues. (**A**–**J**) Histological images of hematoxylin and eosin (H&E) or Masson’s trichrome stained liver tissues collected on days 0, 3, 7, 14 and 28 after the BH treatment. (**A**–**J**) BH treated region. (Scale bar, 1 mm; magnification 50 ×). Images (i) and (ii) show the highlighted areas in (**A** to **J**) at higher magnifications. (Scale bar, 50 µm; magnification 400 ×) CV: central vein, FB: fibroblast, PA: Portal area, and PV: Portal vein.

In our experiments, after the animal’s sacrifice, the BH-treated liver tissue was cross-sectioned and stained with hematoxylin and eosin (H&E) or Masson’s trichrome for histological observation. Partially fractionated and homogenised liver tissue filled with erythrocytes appeared immediately after the BH exposure on day 0 (Fig. 2A and A-ii). No hepatocytes were observed in the BH lesion. Furthermore, several large blood vessels remained intact at the border between the treated and untreated regions as shown in Fig. 2B-i. Three days after the treatment, the BH lesion was filled with early granulation tissue (bright pink colour, Fig. 2C) with increased numbers of proliferating fibroblasts (Fig. 2C-i) and collagen fibers (fibroblast secreting collagen fibers, Fig. 2D-ii). In addition, portal fibroblasts were significantly recruited to the boundary of the BH region (Fig. 2C-ii and D-i). A week after the BH treatment, the formation of small capillaries was observed within the granulation tissue (Fig. 2E-ii and F-ii). From Masson’s trichrome staining results shown in Fig. 2F, a denser collagen matrix with fibroblasts occurred at the boundary of the BH lesion (Fig. 2F-i). On day 14, regenerated hepatocytes and newly formed blood vessels within the BH lesion were found as shown in Fig. 2G-i and H-ii. Some collagen matrix still remained around the portal veins (Fig. 2H and H-i). Along with a significant reduction of collagen matrix, regenerated hepatocytes, portal veins, central veins and portal area were clearly observed within the BH-treated region on day 28 (Fig. 2I and J).

### Degradation of fibrillar collagens by matrix metalloproteinase-2 (MMP-2) activation

**Figure 3:**
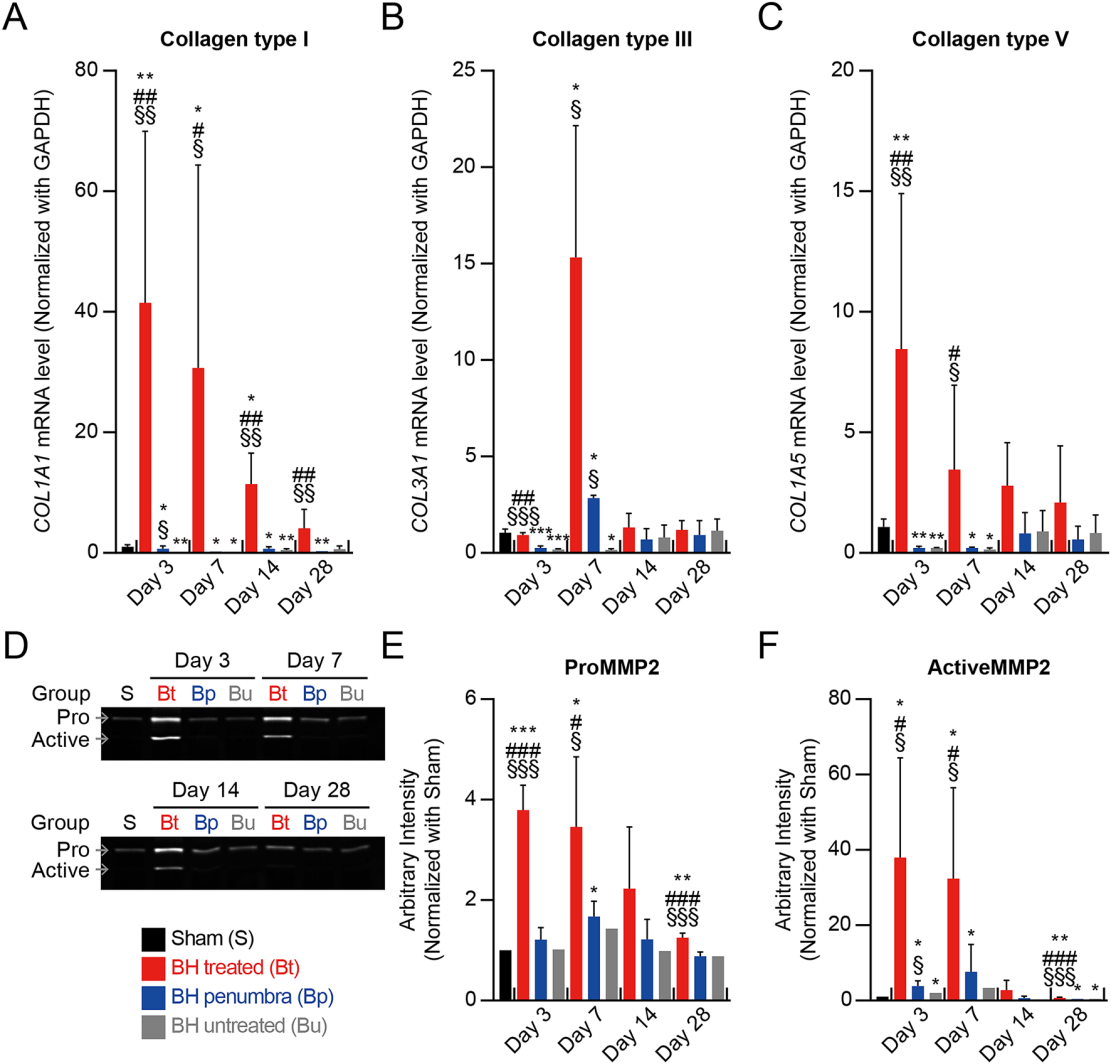
Degradation of fibrillary collagens by active MMP-2. (**A**–**C**) Expression mRNA levels of collagen type I, III and V. *n* = 5 for Sham, *n* = 3 for Day 3 and 7, *n* = 4 for Day 14, and *n* = 5 for Day 28. (**D**) Representative image of MMP-2 gelatin acrylamide gel zymography. (**E** and **F**) Quantitative analysis of the relative levels of MMP-2 activity in liver lysates. *n* = 2 for Sham, *n* = 3 for Day 3, *n* = 4 for Day 7 and 14, and *n* = 5 for Day 28. Data are presented as means ± SD. One-way ANOVA followed by post-hoc Tukey’s test was used. ^*§*^*p* < *0.05 vs. BH untreated of each time point, *^*§§*^*p* < *0.01 vs. BH untreated of each time point, *^*§§§*^*p* < *0.001 vs. BH untreated of each time point, *^*#*^*p* < *0.05 vs. BH penumbra of each time point, *^*##*^*p* < *0.01 vs. BH penumbra of each time point, *^*###*^*p* < *0.001 vs. BH penumbra of each time*, *p < *0.05 vs. sham*, **p < *0.01 vs. sham* and ***p < *0.001 vs. sham*.

MMP-2 (gelatinase A) is a key representative inhibitor of collagen. Excessive collagen formation after injury is regulated by MMP-2 activation which breaks down the collagens^33^. In the present study, qPCR assay for measurement of collagen expression (collagen type I, III and V) and gelatin zymography for detection of MMP-2 activation were performed on the BH-treated, BH-penumbra and BH-untreated regions (Fig. 3 and Supplementary Fig. S1). On day 3, the mRNA expression level of collagen type I (the most abundant protein among the fibrillar collagens of ECM) at the BH-treated region was 41-fold higher than that of the sham group, as shown in Fig. 3A. Along with this, significant elevations of pro and active MMP-2 levels were also observed (i.e., 3.8-fold change in proMMP-2 and 38-fold change in activeMMP-2) (Fig. 3D–F). These were then reduced on day 7 and reached a base level on day 28. These findings indicate the breakdown of the ECM structure under activation of MMP-2. Similarly, collagen type III that is one of the major components of early granulation tissue was upregulated to 15-fold change on day 7 and gradually reduced to a base level on day 14 (Fig. 3F). The formation of granulation tissue was histologically confirmed on day 7 (Fig. 2E). Lastly, the mRNA level of collagen type V, which is regulatory fibril-forming collagen^34^, was upregulated by sevenfold on day 3 and started to decrease from day 7 (Fig. 3C). On contrary, during the experiments, no significant changes of collagen type I, III, V and MMP-2 levels were observed at the BH-penumbra region. These were similar to those measured at the untreated region.

### Apoptosis of fibroblast and damaged hepatocytes

**Figure 4:**
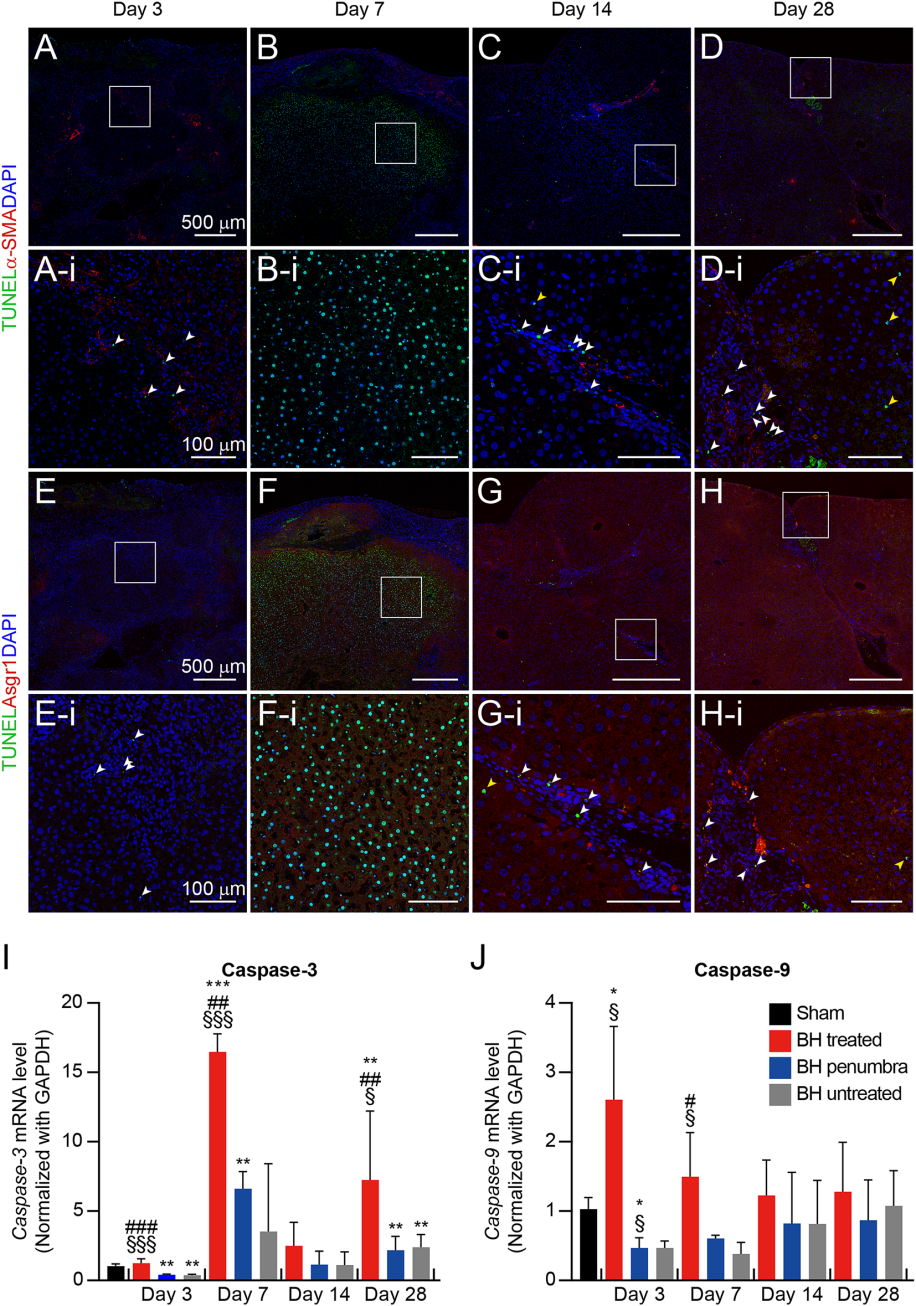
Cellular apoptosis in BH-treated liver tissues. (**A**–**D**) Representative IHC staining images of apoptotic fibroblasts in cross-sectioned BH-treated livers (green, TUNEL staining, red, α-SMA, blue, DAPI). (**E**–**H**) Representative IHC staining images of apoptotic hepatocytes in BH-treated region (TUNEL staining; green, Asgr1; red, DAPI; blue). (Scale bar, 500 µm; magnification 100 ×) (A-*i*–*H-i*) Magnified representative images for co-localization of TUNEL staining (green) and DAPI (blue) in cross-sectioned BH-treated livers. Co-localized fibroblasts (white arrowheads) and hepatocytes (yellow arrowheads). Nuclei were counterstained with DAPI. (**I** and **J**) Expression mRNA levels of caspase-3 and caspase-9. *n* = 5 for Sham, *n* = 3 for Day 3 and 7, *n* = 4 for Day 14, and *n* = 5 for Day 28. Data are presented as means ± SD. One-way ANOVA followed by a post-hoc Tukey’s test was used. ^*§*^*p* < *0.05 vs. BH untreated of each time point, *^*§§*^*p* < *0.01 vs. BH untreated of each time point, *^*§§§*^*p* < *0.001 vs. BH untreated of each time point, *^*#*^*p* < *0.05 vs. BH penumbra of each time point, *^*##*^*p* < *0.01 vs. BH penumbra of each time point, *^*###*^*p* < *0.001 vs. BH penumbra of each time*, *p < *0.05 vs. sham*, **p < *0.01 vs. sham* and ***p < *0.001 vs. sham*.

Protease enzymes caspase-3 and -9 are responsible for apoptosis that is programmed cell death. Caspase-9 can be activated by injury and promote the activation of caspase-3 that destructs cellular structure^35,36^. In the wound healing process, the activation of caspase enzymes is the important biochemical change of apoptosis which results in cessation of collagen accumulation. In the present study, the induction of apoptosis in the BH-treated liver tissue was observed through triple immunohistochemistry (IHC) staining and the mRNA level of caspase enzymes. Three days after the BH treatment, only a few fluorescent signals of terminal deoxynucleotidyl transferase dUTP nick end labeling (TUNEL)-positive apoptotic fibroblasts (α-SMA) and hepatocytes (Asgr1) were observed in the whole BH-treated tissue region (Fig. 4A,A-i,E,E-i and Supplementary Figs. S2 and S3). On day 7, most TUNEL-positive apoptotic cells were hepatocytes (Fig. 4F,F-i and Supplementary Fig. S3), while apoptotic fibroblasts were rarely observed (Fig. 4B,B-i and Supplementary Fig. S2). However, on days 14 and 28, the number of apoptotic hepatocytes was significantly reduced, compared to that observed on day 7 (indicated by the yellow arrowheads in Fig. 4G,G-i,H,H-i, and Supplementary Fig. S3), whereas apoptotic fibroblasts were persistently found (indicated by the white arrowheads Fig. 4C,C-i and D-i, and Supplementary Fig. S2). The changes of mRNA level of caspase-3 can represent the changes in apoptotic cell population in the BH-treated liver tissue (Fig. 4I). The mRNA level of caspase-3 was a 16-fold change on day 7, which resembled the increased TUNEL-positive apoptotic hepatocytes observed in the BH-treated lesion at the identical time point (Fig. 4F,F-i and I). Similarly, elevated caspase-3 mRNA level on day 28 can support the presence of apoptotic fibroblasts on day 28 (Fig. 4D,D-i and I). The mRNA level of caspase-9 reached the maximum on day 3 and gradually reduced to a base level on day 14 (Fig. 4J). These results suggested that the BH-induced apoptosis of hepatocytes reached the maximum on day 7 and then was diminished while apoptotic fibroblasts were continuously observed on day 28.

### Normal gaining weight and normal recovery of ALT and AST levels after the BH treatment

**Figure 5:**
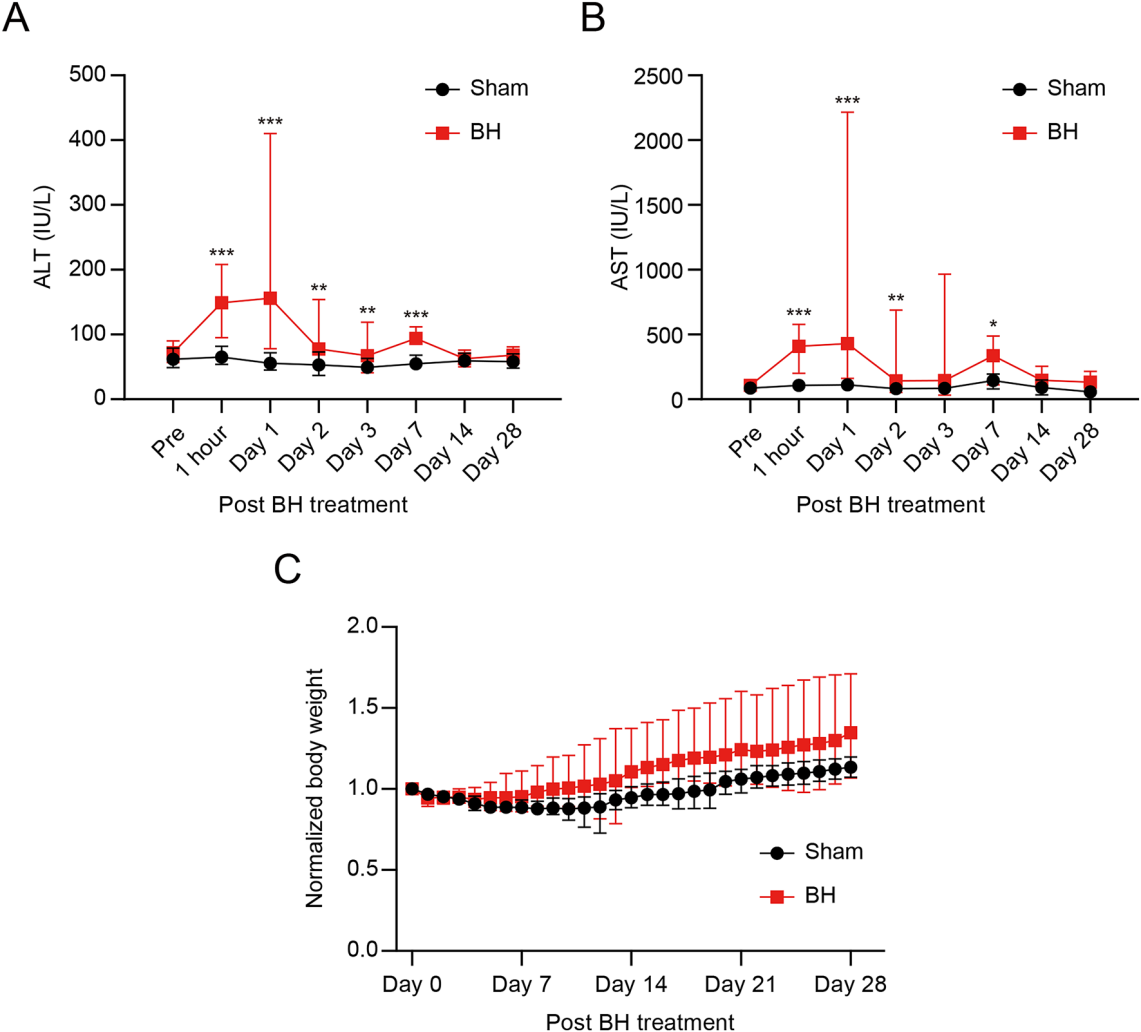
Recovery of surrogate markers of liver injury and body weight after BH-treatment. (**A** and **B**) Changes in serum AST and ALT in response to BH-treatment. Pre: prior to BH-treatment. *n* = 16 for Pre, *n* = 19 for 1 h, Day 1, 2, and 3, *n* = 4 for Day 7 and 14, *n* = 5 for Day 28. Data are presented as means ± SD. One-way ANOVA followed by a post-hoc Tukey’s test was used. *p < *0.05*, **p < *0.01* and ***p < *0.001.* (**C**) Changes in normalised body weight after BH treatment. Body weights were normalised with average body weight of Day 0. *n* = 36 for Day 0, 1, 2, and 3, *n* = 29 for Day 4, 5, 6, and 7, *n* = 20 for Day 8 and 9, *n* = 18 for Day 10, 11, 12, and 13, *n* = 11 for Day 14, *n* = 9 for Day 15 to 28. Data are presented as means ± SD. One-way ANOVA followed by a post-hoc Tukey’s test was used. No significant change was observed.

Blood samples were collected from the BH-treated rats (*n* = 19) at 1 h and 1, 2, 3, 7, 14, and 28 days after the BH treatment. For comparison purpose, blood samples from the sham group (i.e., no BH exposure) were also obtained. The mean serum ALT and AST levels in the sham did not significantly change (ALT of 57 IU/L and AST of 95 IU/L), whereas both serum ALT and AST levels of the BH-treated group drastically increased to 149 IU/L and 408 IU/L respectively at 1 h, as shown in Fig. 5A,B. These values were maintained until day 1 and then decreased to 74 IU/L (ALT) and 102 IU/L (AST) on day 2. No significant changes in the body weight of the BH-treated rats were observed (Fig. 5C and Supplementary Fig. S4).

## Discussion

In the present study, for the first time in BH fields, we extensively investigated the long-term wound healing process of liver tissue after the BH exposure. In general, when liver tissue is injured, hemostasis occurs first. Neutrophils and macrophages then produce pro-inflammatory cytokines resulting in myofibroblast-transdifferentiation by hepatic stellate cell activation and resident fibroblasts-acquiring myofibroblast phenotype^37^. Activated resident fibroblast and myofibroblast are essential regulators in the formation of granulation tissue which is involved in the maintenance of physical integrity of ECM structure and the closure of the wounded tissue. It is worth noting that a healthy granulation tissue that consists of capillaries, immune cells and fibroblasts plays a prominent role in wound repair of the supplement of nutrients, protection of healing tissue and formation of dense ECM^38,39^. Activated fibroblasts migrate from portal vein to the wound site and then secrete collagenous fibers during inflammation and proliferation phases^37^. This process eventually results in the formation of granulation tissue. During the maturation phase, myofibroblasts produce decorin inducing cytokine degradation whereby activation of fibroblast is reduced and the inactivated myofibroblasts undergo apoptosis^40^. In the liver, ECM structure formed after injury as a part of healing process can be broken down by MMPs expression^33^ and then hepatocytes can subsequently be regenerated at the injury site. In these contexts, we observed that the BH lesion was rapidly populated by early granulation tissue including ECM structure for the first three days after the BH exposure. New capillaries were generated in the granulation tissue structure (Fig. 2E-ii and F-ii). This is of paramount importance as the new blood vessels can provide nutrients and oxygen to regenerated hepatocytes in the BH lesion^41,42^. The amount of collagen type III in the granulation tissue increased on day 7 (Fig. 3B) and then returned to a normal value due to degradation of collagens by upregulated MMPs as well as replacement of collagen type III of a fragile structure to collagen type I of denser structure^40,43^.

Our experimental results clearly demonstrated the feasibility of inducing localised biological effects by BH (Figs. 1C and 3A–C). The expressions of collagens and α-SMA at the BH penumbra were similar to those at the BH-untreated region and the sham group whilst those at the BH-treated region drastically increased. In addition, a number of intact blood vessels and hepatocytes were observed near the boundary of the BH-treated region (Fig. 2B-i and D-i). The activated portal fibroblasts migrated to the BH region through these intact blood vessels resulting in the construction of granulation tissue including ECM (Fig. 2C–F).

In case of chronic liver injury (e.g., alcoholic liver disease, infection of hepatitis B and C viruses), the presence of hepatic fibrosis by excessive ECM deposition can lead to liver cirrhosis and hepatocellular carcinoma^44^. In contrast, for acute liver damage (e.g., injection of chemical hepatotoxins such as acetaminophen and carbon tetrachloride), damaged hepatocytes can be replaced by proliferating hepatocytes through liver regeneration process including the cessation of fiber production by fibroblast apoptosis and collagen matrix breakdown by the up-regulated MMPs^44^. In the present study, two weeks after the BH exposure, the BH-treated liver tissue was filled with regenerated hepatocytes without the presence of hepatic fibrosis (Fig. 2I-ii). Angiogenesis was also observed on days 14 and 28 according to the histological analyses shown in Fig. 2H-ii and J-i. On day 28, mRNA expression of collagens in the BH treated region exhibited 3.5 fold in type I, 1.08 fold in type III, and 1.79 fold in type V compared to those observed in the sham group (Fig. 3A–C). In general, the elevation of ALT and AST can be found in the bloodstream when hepatocyte damage occurs^45,46^. In the serological tests performed, the serum AST and ALT levels dramatically increased on day 1 after the BH treatment due to the death of hepatocytes (Fig. 5A and B). Subsequently, these enzymes returned to a base level. In addition, during our experiments, the body weight of the BH treated rats slightly increased over time; however, no adverse tissue effects such as steatosis, chronic fibrosis and enlarged or ballooned hepatocyte were histologically observed (Fig. 2).

In conclusion, to the best of our knowledge, this is the first study reporting the long term investigation of the wound healing process of liver after BH treatment in vivo. Our experimental results clearly showed that BH can trigger normal wound healing cascade and tissue regeneration (e.g., ECM remodeling, angiogenesis, hepatocyte regeneration) without inducing hepatic fibrosis or forming permanent scar tissue at the treatment site. These can clearly suggest that BH technique could be a promising and safe therapeutic tool for liver cancer treatment.

## Methods

In the present study, BH was applied to produce multiple volumetric lesions on a rat’s liver in vivo. Upon animal sacrifice at 0, 3, 7, 14 and 28 days after BH treatment, BH-treated liver tissue and blood samples were collected for morphological, histological, serological and quantitative real-time polymerase chain reaction (qPCR) analyses to investigate the changes of the extent of extracellular matrix (ECM) remodeling, mRNA expression of cells (hepatocytes and fibroblasts), apoptosis status and serum aspartate aminotransferase (AST) and alanine aminotransferase (ALT) levels over time.

### Animal experimentation

Six weeks old male SD rats were obtained from Koatech (Pyeongtaek, Korea). All rats were housed under a 12-h light/dark cycle with free access to water and food. All experimental protocols were approved by the Institutional Animal Care and Use Committee of Korea University College of Medicine (Approval No. KOREA-2018-0030). All the methods were performed in accordance with the relevant guidelines and regulations.

### Surgery

After 2-weeks of acclimation, general anesthesia was performed with 3.5% isoflurane in a 2:1 N2O/O2 mixture. The mixture of gas was maintained in the anesthesia chamber via rat’s inhalation through a 2.5% nasal cone. Rats were placed on the warm pad and subjected to the midline xipho-pubic laparotomy, as previously described in^47^. Both peritoneum and xiphoid were bent and pushed aside using silk surgical sutures to expose the left lateral lobe of the liver. Sterile gauze was located between the liver and stomach to remove the blood and prevent potential gastrointestinal damage associated with BH exposure. After BH exposure, rats were closed by layers with 4-0 sutures and allowed free access to water and food upon wakening.

### HIFU experimental setup and exposure conditions

Prior to BH exposure, the rat’s liver was partially exteriorised to simplify the guidance of the HIFU focus, as performed in^16^. A photograph of the HIFU experimental setup employed in the present study is shown in Supplementary Fig. S5. A 2-MHz single element HIFU transducer (H148, Sonic Concepts, USA) with an aperture size of 64 mm, a focal length of 63.2 mm and lateral and axial full width half maximum dimensions of 7.25 mm and 0.89 mm was used to produce a number of BH lesions in the liver. The HIFU source was driven by a function generator (33600A, Keysight Technologies, USA) and a power amplifier (1040L, Electronics & Innovation, USA). During the experiments, a customised cone-shaped transducer holder which was filled with degassed water and connected to a manual-three-axis-positioning system (SD3-118-L5W, Stage Number 1, Republic of Korea) was placed on the animal’s exteriorised left lateral lobe of liver. The surface area of the partially exteriorised liver was about 2.25 cm^2^ (1.5 cm × 1.5 cm). A 12-μm thick acoustically transparent Mylar film (PMX980, HiFi Industrial Film, UK) was attached to the end of the holder for coupling purpose. This transducer holder was designed so that the distance from the tip of the holder to the centre of the transducer surface was 60.2 mm, and whereby the HIFU focus was 3 mm below the surface of the liver. A laser pointer aligned with the HIFU transducer’s axial axis was attached to the translation stage for targeting the HIFU beam on the exteriorised liver surface laterally. BH lesions were produced by raster-scanning the transducer focus over the area of 1.44 cm^2^ using 2 mm steps in the transverse and lateral directions (up to a maximum of 1.2 cm), as illustrated in Fig. 1A. A number of multiple BH lesions were deliberately produced in order to trigger a sufficient healing response of the liver. A total number of 10 BH pulses each containing a 10 ms-long HIFU burst with peak positive and negative pressures of 85 MPa and—14 MPa (16) were delivered at each focus with a duty cycle of 1% and a pulse repetition frequency of 1 Hz. The BH exposure conditions used in the present study have been widely used in the previous BH studies^13,14,23^. After the BH treatment, the animals were placed back into the animal housing facility and were sacrificed on day 3 (*n* = 3), 7 (*n* = 2), 14 (*n* = 4), and 28 (*n* = 5). Upon euthanisation, the liver tissues containing BH-treated, BH-penumbra (i.e., the area surrounding the BH-treated region) and BH-untreated regions (Supplementary Fig. S1) and blood samples were collected for analyses.

### Determination of the BH exposure conditions used in the present study

The effect of varying the number of BH pulses (1, 5 and 10 pulses) on the degree of mechanical damage produced in the liver was initially investigated in vivo (Supplementary Fig. S6). A positive relationship between the number of BH pulses and the degree of mechanical destruction was observed^9,10,13^. With a single BH pulse, a low degree of damage without destruction of blood vessels was induced, as shown in Supplementary Fig. S6A,B. Five BH pulses resulted in partial fractionation of liver tissue (Supplementary Fig. S6C,D). Cell debris was also observed; however, no blood vessels were damaged (Supplementary Fig. S6C-i and D-i). With 10 BH pulses, liver tissue at the HIFU focus was fractionated which was filled with erythrocytes (Supplementary Fig. S6E,F). A few blood vessels (capillaries) were also destructed due to the high degree of damage produced by BH (Supplementary Fig. S6F-i). The destructed liver tissue size with 10 BH pulses was approximately measured to be 1 mm (width) × 4.5 mm (length). In the present study, therefore, 10 BH pulses were used to study a long-term wound healing process of the liver after BH treatment.

### Histopathology

Rats were humanely sacrificed after 3, 7, 14, and 28 days after BH exposure by using the CO_2_ chamber. After sacrifice, BH-treated rat liver tissues were immediately collected and placed in 4% paraformaldehyde (PFA, Biosesang) for histological examination. The tissues were fixed in 4% PFA for 48 h and after dehydration in 70% ethanol, embedded in a paraffin block. Following the standard protocols^48^, the paraffin-embedded liver tissue was cut in 4.5 μm thickness and stained with H&E and MT. Next, stained sections were imaged using the EVOS m7000 imaging system (Thermo Fisher Scientific) and Zeiss Axio Scan Z1 (Carl Zeiss).

### Immunohistochemistry

After deparaffinization and rehydration, liver sections were incubated with the following primary antibodies: anti-asialoglycoprotein receptor 1/HL-1 (ASGR1) antibody (1:200 dilution, ab127896, Abcam), and anti-α-SMA (1:400 dilution, ab7817, Abcam). Anti-goat IgG Alexa Fluor 488 and 594 (Invitrogen, Carlsbad, CA, USA) were used for the secondary antibody. All fluorescence images were obtained with LSM 800 (Carl Zeiss) and Zeiss Axio Scan Z1 (Carl Zeiss).

### In situ* detection of apoptotic cells*

Apoptotic cells in BH-treated liver sections were analyzed using a TUNEL in situ apoptosis detection kit (Dead-End Colorimetric TUNEL system, Promega, Madison). The assay was conducted according to the manufacturer’s instructions. Counterstaining was performed with 4′,6-diamidino-2-phenylindole (DAPI) and anti-α-SMA (Abcam) or anti-ASGR1 antibody labeled with anti-goat IgG Alexa Fluor 594 (Invitrogen, USA).

### Blood analysis

Prior to BH treatment, femoral artery cannulation was performed, as previously described in^47^. Blood samples were collected at the following intervals separately; 10 min before BH-treatment, 1 h, 24 h, 48 h, 72 h after BH-treatment, and upon sacrifice. Using a FUJI DRI-Chemiclinical Chemistry Analyzer (FUJI DRI-CHEM 4000i, Fuji Film), the levels of ALT (alanine aminotransferase) and AST (aspartate aminotransferase) in serum were measured.

### Quantitative real-time RT-PCR (qRT-PCR)

Liver tissues for qRT-PCR were collected from regions labelled in Supplementary Fig. S1. Total RNA from liver tissues extracted with the Trizol reagent (Invitrogen). Using the reverse transcription reaction kit (iScript cDNA synthesis kit, Bio-Rad), the cDNA was synthesis from 1 μg of total extracted RNA. The measurement of gene expression was performed with the SYBR Green mixture (iQTM SYBR Green Supermix, Bio-Rad) and the iCycler PCR thermocycler (Bio-Rad). Specific Primers for target gene mRNA were designed using the Real-time PCR (TaqMan) Primer and Probes Design Tool (GenScript, Piscataway). The mRNA levels were normalized with the mRNA level of glyceraldehyde 3-phosphate dehydrogenase (GAPDH), as previously reported^49^. The sequence of template-specific primers were as follows: Caspase3 (5’-TTTGGAACGAACGGACCTGT-3’ and 5’- GGCAGGCCTGAATGATGAAG-3’), Caspaes9 (5’-AAGCAGGATCCAGAAGCTGT-3’ and 5’- GAGATGGGTCCAGCTTCACT-3’), COL1A1 (5’-GATCTCCTGGTGCTGATGGA-3’ and 5’- GACCAGGGAAGCCTCTTTCT-3’), COL3A1 (5’-ACTGGTGAACGTGGCTCTAA-3’ and 5’- GGACCTGGATGTCCACTTGA-3’), COL5A1 (5’-CTTCGGGAGCAGATGGTGAA-3’ and 5’- CTGGAGGTCCTGGTAAACCC-3’), GAPDH (5’-CAAGGCTGAGAATGGGAAGC-3’ and 5’-GAAGACGCCAGTAGACTCCA-3’), and αSMA (5’-GCTATTCAGGCTGTGCTGTC-3’ and 5’- GTTGTGAGTCACGCCATCTC-3’).

### Gelatin zymography

Liver tissues for gelatin zymography were collected from regions labelled in Supplementary Fig. S1. The activity of pro-MMP-2 and active MMP-2 in BH-treated liver tissues was assessed with gelatin zymography. Livers obtained from sacrificed rats were harvested in 0.9% saline and lysed in RIPA buffer (Thermo Fisher Scientific) with protease inhibitor (Gendepot, Katy, TX). The protein concentration in the liver lysate was measured with the BCA protein assay kit (Thermo Fisher Scientific). Each lysate containing 10 μg of protein was mixed with a non-reducing Laemmli sample buffer and transferred to 10% gelatin acrylamide gels without heating. The gels consisted of 1 mg/mL gelatin powder (JT Baker Chemical Co.) and 0.4% glycerol (Sigma-Aldrich) in 10% SDS-PAGE gel with conventional composition. Liver lysates were separated by electrophoresis at 175 V for 120 min. As a positive control, 10 μg of MMP-2 recombinant protein (ab81550, Abcam) was loaded. After gel electrophoresis, the gels were incubated in zymogram renaturing buffer (Invitrogen) at room temperature with shaking for 2 h. Subsequently, gels were immersed in zymogram developing buffer (Invitrogen) for 30 min at room temperature with shaking. Then gels were transferred to a fresh zymogram developing buffer and incubated for 48 h at 37 °C with shaking. Gels were stained with a colloidal blue staining kit (Invitrogen), and images of stained gels were captured with a Bio 5000 scanner (Microtek). The activity of pro-MMP-2 and active MMP-2 was analyzed with ImageJ open-source software^50^.

### Statistical analysis

All data were expressed as mean ± standard deviation. Shapiro–Wilk test was performed to evaluate normality distribution. One-way analysis of variance (ANOVA) followed by post-hoc Tukey’s correction was used for parametric analysis and the Kruskal–Wallis test with a post-hoc Conover correction for non-parametric analysis. Data were analyzed using MedCalc version 20.008 (MedCalc, Mariakerke, Belgium). A p-value of < 0.05 was considered statistically significant for all analyses.

### Ethical approval

This study was reported in accordance with ARRIVE guidelines.

## Supplementary Information

Supplementary Information.

## Data Availability

All study data are included in this manuscript and Supplementary information. All the experimental data presented in this study is available upon request. Contact Ki Joo Pahk to request the data.
