# Supplementary material for: Investigation of the long-term healing response of the liver to boiling histotripsy treatment in vivo
Source: Sci Rep. 2022 Aug 24;12:14462. doi: 10.1038/s41598-022-18544-7 (PMC9402918; doi:10.1038/s41598-022-18544-7)
Supplement: Supplementary file 1 — Supplementary Information. [file 41598_2022_18544_MOESM1_ESM.docx]

**Supplementary Information**

**Title*:*** Investigation of the long-term healing response of the liver to boiling histotripsy treatment in vivo

**Authors Names**

Jeongmin Heo^a,#^, Chanmin Joung^b,#^, Kisoo Pahk^c,*^, Ki Joo Pahk^d,*^

^a^Center for Bionics, Biomedical Research Institute, Korea Institute of Science and Technology (KIST), Seoul, Republic of Korea

^b^Institute for Inflammation Control, Korea University, Seoul, Republic of Korea

^c^Department of Nuclear Medicine, Korea University College of Medicine, Seoul, Republic of Korea

^d^Department of Biomedical Engineering, Kyung Hee University, Yongin, Republic of Korea.

^#^Contributed equally to this work

*Corresponding authors

**Corresponding authors**

Ki Joo Pahk

Department of Biomedical Engineering, Kyung Hee University, 1732 Deogyeong-daero, Giheung-gu, Yongin-si, Gyeonggi-do, Republic of Korea 17104.

+82(0)31-201-2572

kjpahk@khu.ac.kr

Kisoo Pahk

Department of Nuclear Medicine, Korea University College of Medicine, Anam-dong 5-ga, Seongbuk-gu, Seoul, Republic of Korea 02841.

kisu99@korea.ac.kr


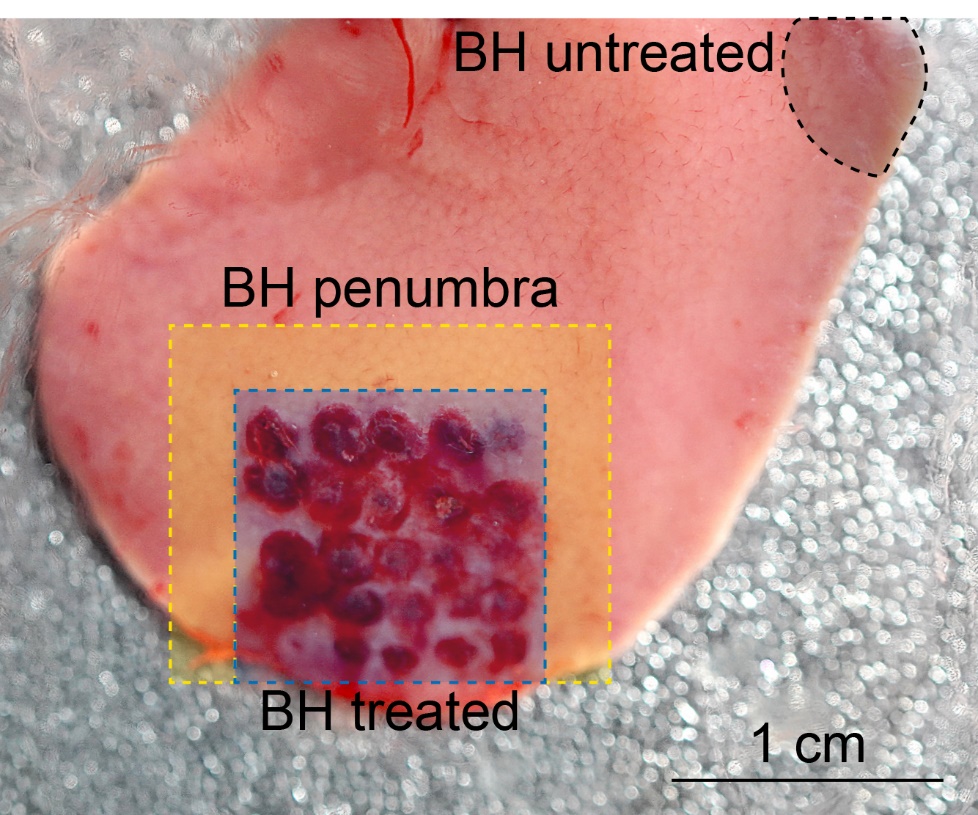


Supplementary Fig. S1. BH treated, BH penumbra and BH untreated regions used in the analyses performed in the present study.


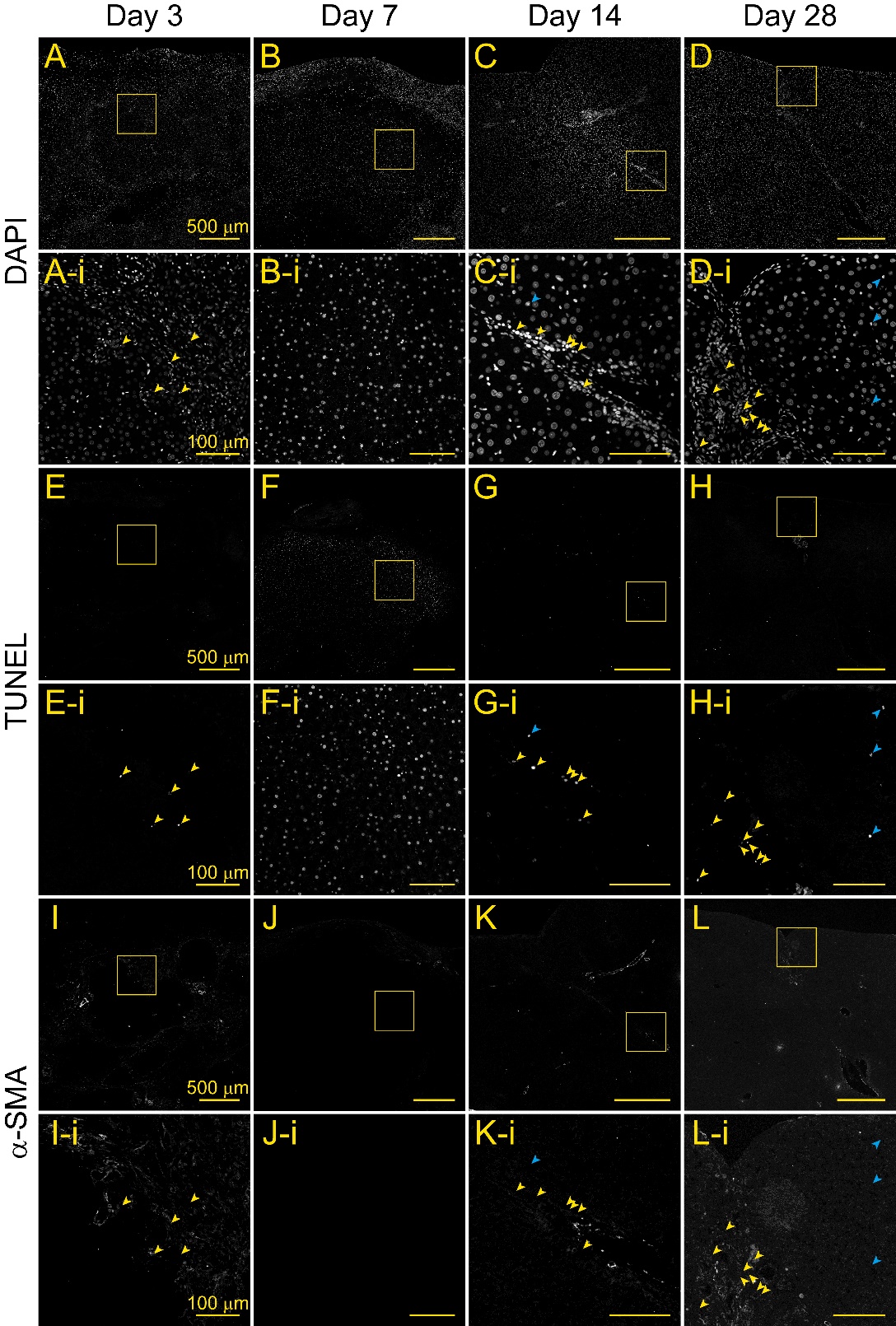


I

K

E

Supplementary Fig. S2. Effect of the BH treatment on fibroblasts apoptosis in liver tissues. (*A* – *L*) Representative images of apoptotic fibroblasts in cross-sectioned BH-treated livers. (Scale bar, 500 µm; magnification 100x) (A-*i* – *L-i*) Magnified representative images of apoptotic fibroblasts in cross-sectioned BH-treated livers. The yellow and blue arrowheads indicate apoptotic fibroblasts and hepatocytes, respectively. (Scale bar, 100 µm; magnification 400x)


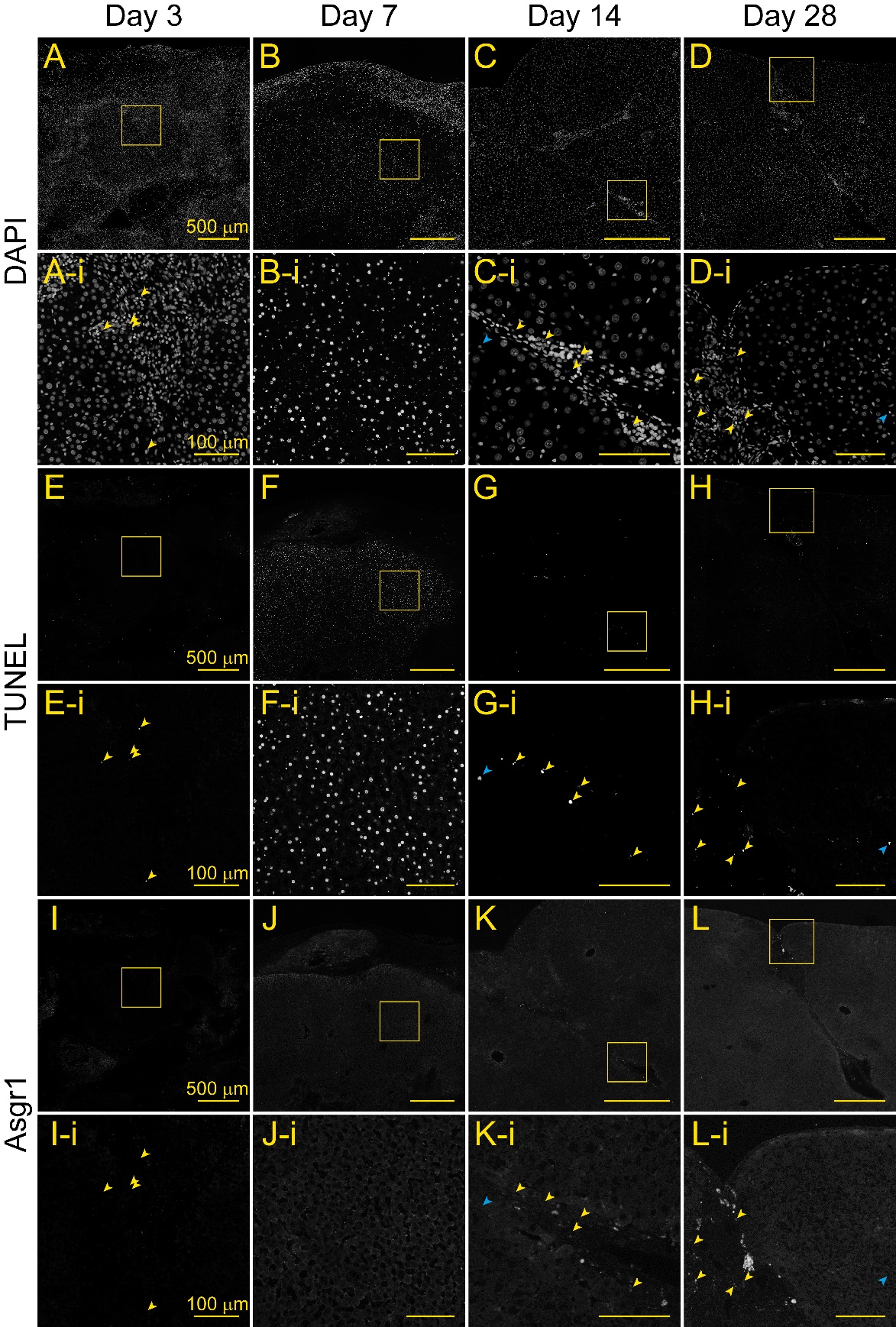


Supplementary Fig. S3. Effect of the BH treatment on hepatocyte apoptosis in liver tissues. (A – L) Representative images of apoptotic fibroblasts in cross-sectioned BH-treated livers. (Scale bar, 500 µm; magnification 100x) (A-i – L-i) magnified representative images of apoptotic fibroblasts in cross-sectioned BH-treated livers. Apoptotic fibroblasts (yellow arrowheads) and hepatocytes (blue arrowheads). (Scale bar, 100 µm; magnification 400x)


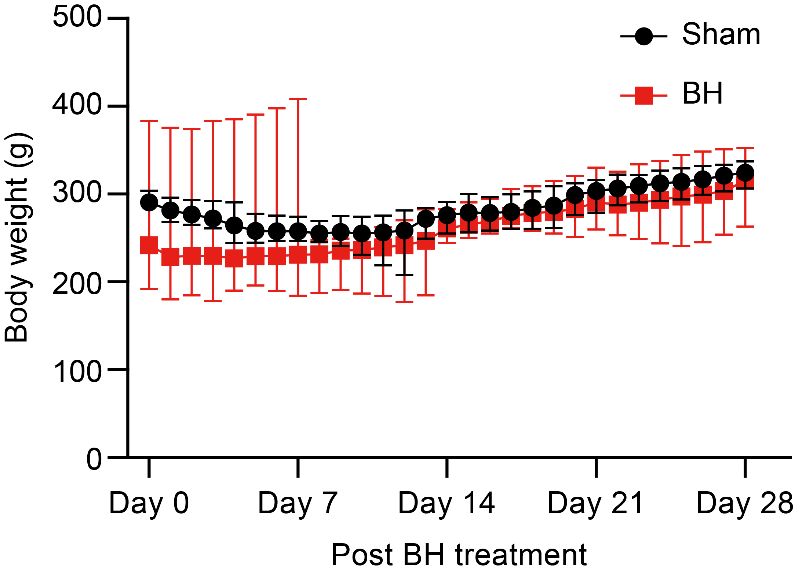


Supplementary Fig. S4. Changes in body weight after BH treatment


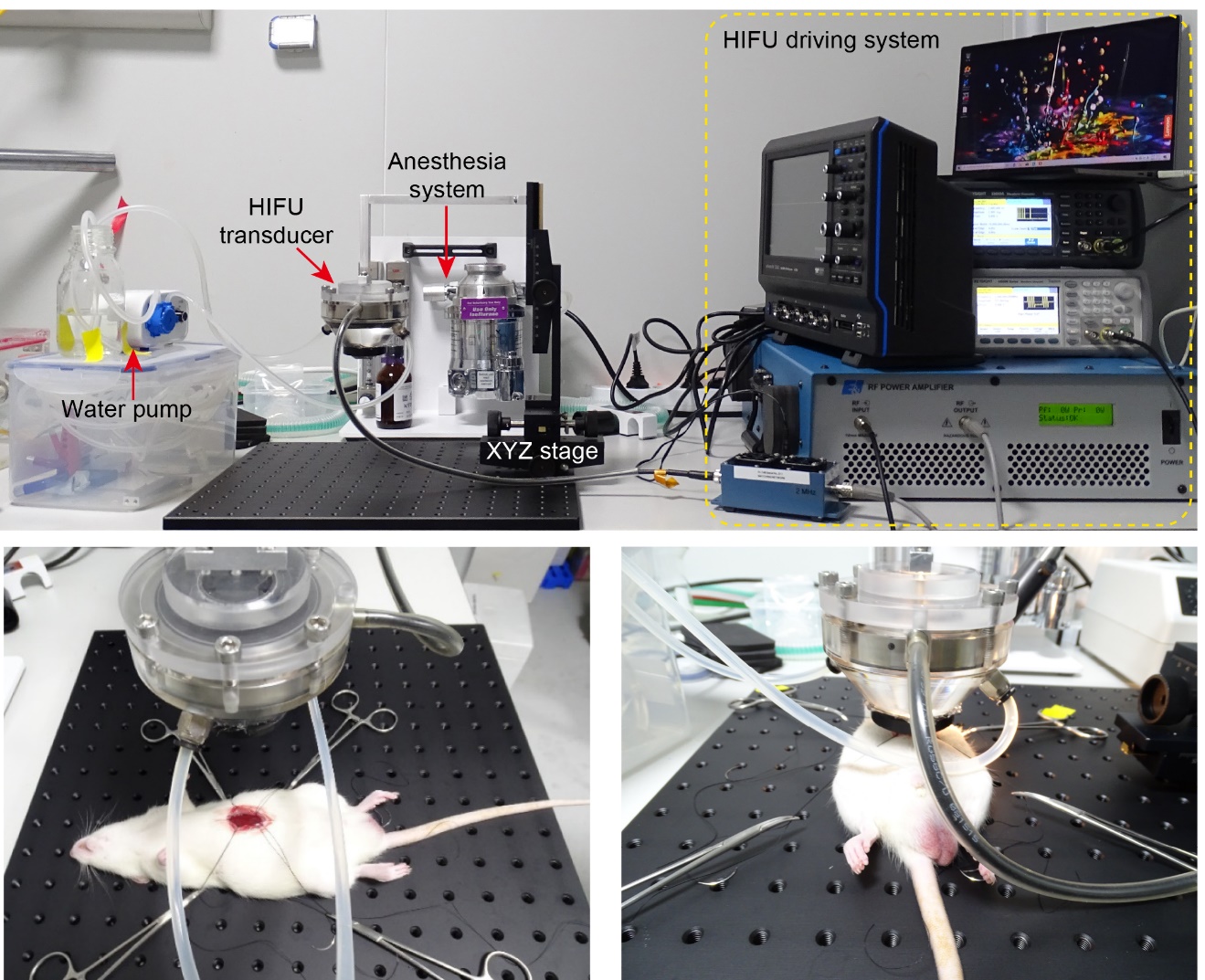


Supplementary Fig. S5. In vivo experimental setup used for performing boiling histotripsy in rat’s exteriorised liver.


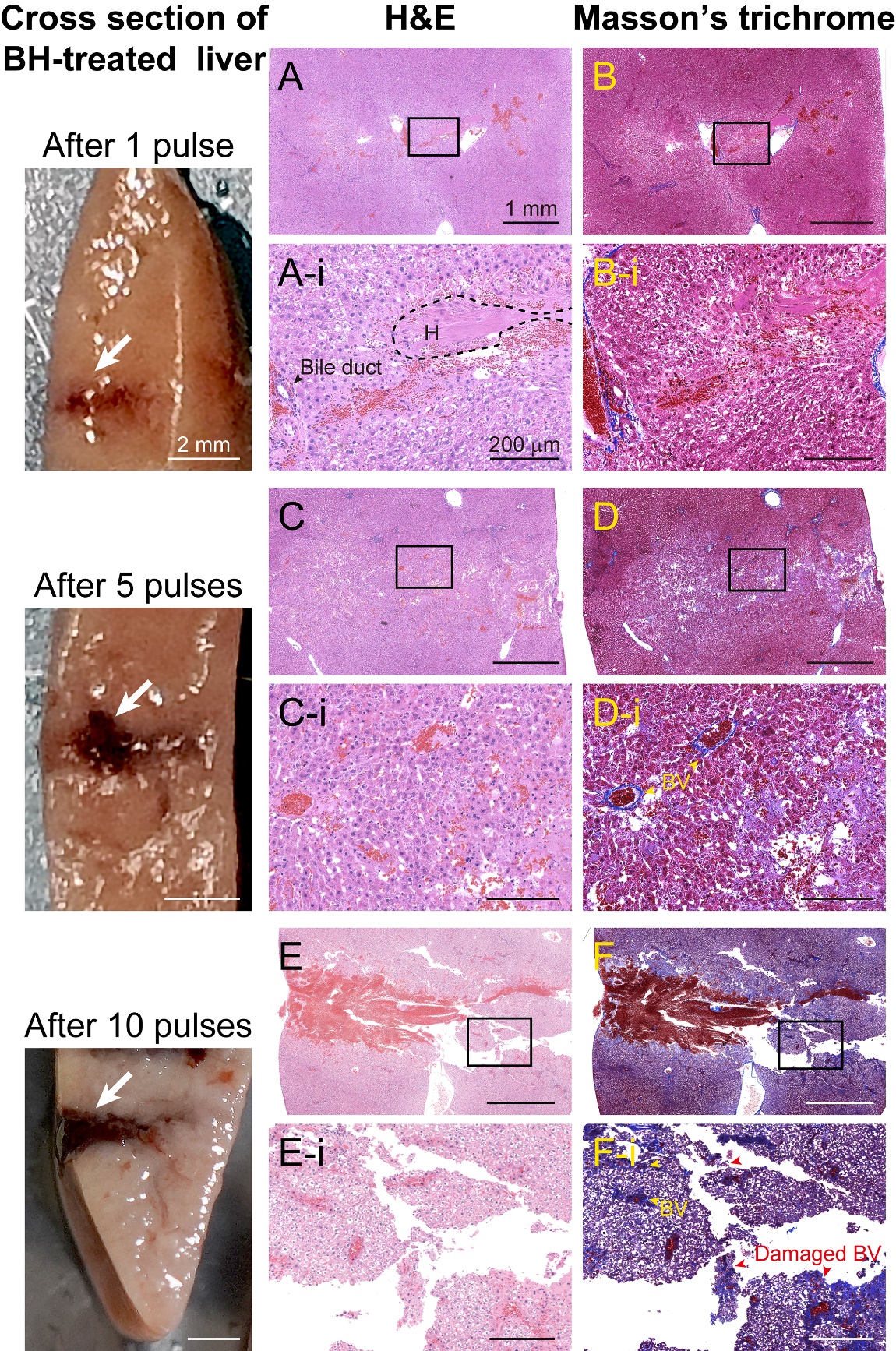


Supplementary Fig. S6. Representative cross-sectional images of boiling histotripsy lesions produced with 1, 5, or 10 pulses. (*A* – *F*) Histological images of H&E and Masson’s trichrome staining. (Scale bar, 1 mm; magnification, 50x). (*A-i* – *F-i*) magnified images of the highlighted areas in in (*A* – *F*). (Scale bar, 200 µm; magnification, 200x) H: homogenised tissue, BV: blood vessel.


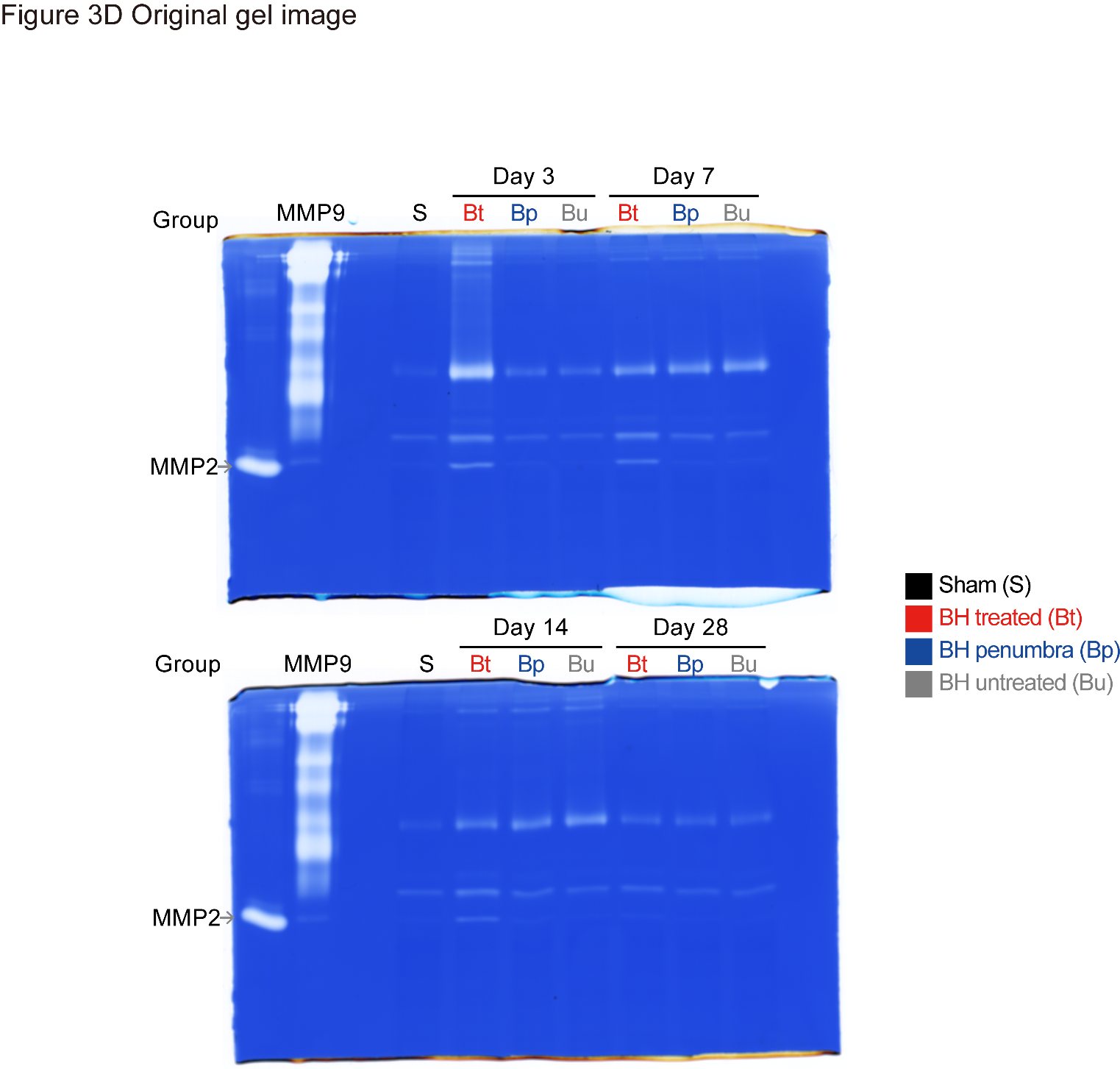


Supplementary Fig. S7. Original gel image of Figure 3D.
